# Parastomal hernia mesh repair, variant of surgical technique without stoma relocation

**Published:** 2012-06-18

**Authors:** P Guriţă, R Popa, B Bălălău, R Scăunaşu

**Affiliations:** * General Surgery Department, “Sf. Pantelimon” Emergency Hospital, Bucharest; ** "Carol Davila” University of Medicine and Pharmacy, Bucharest; General Surgery Department, “Sf. Pantelimon” Emergency Hospital, Bucharest; *** General Surgery Department, „Colţea” Clinical Hospital, Bucharest

**Keywords:** parastomal hernia, minimal invasive, alloplastic procedure, quality of life

## Abstract

**Rationale:**Due to the improvement of prognosis through adjuvant therapy, the life expectancy of neoplasia patients is continuously increasing, which, in conjunction with the progressive occurrence of parastomal hernias during the disease evolution, explains the growing number of reported parastomal hernias affecting patients with permanent colostomy.

Conventional techniques of local repair are inappropriate considering the high recurrence rate, and the decision of stoma relocation depends on the associated pathology, which may counter-indicate general anesthesia, and on previous surgical interventions that are usually followed by a dense peritoneal adhesion syndrome .

**Objective:**The purpose of this article is to make known a variant of alloplastic technique, without translocation, with a low degree of invasiveness, which can be performed successfully under spinal anesthesia, followed by a reduced period of hospitalization.

**Methods and Results:**The study group consisted of 6 patients with permanent left iliac anus who underwent these interventions one to three years prior to the occurrence of parastomal hernia.

Patients were followed at 1 year and 2 years postoperatively and the results were favorable, with no recurrence and improved quality of life through proper prosthesis of the stoma

**Discussion:**We suggest that this technique variation is applied to small and medium parastomal hernias, in case of patients with permanent left iliac anus, with the declared intent of minimal invasiveness.

## Introduction

Colostomy represents a solution, which is frequently used in colorectal surgery, as a mandatory gesture in surgical techniques such as rectum amputation or Hartmann’s operation. The surgical technique variants are well known and can be performed in open or laparoscopic surgery [**11,14**].

Stomas are often followed by complications, some serious and difficult to solve, the parastomal hernia being the most common [**8**]. Its incidence, although difficult to quantify, varies in very large limits, depending on the studied groups or the diagnostic method used, and can reach impressive rates of 78% in ten years.

**Table 1:** Studies of parastomal hernia incidence

STUDY	PARASTOMAL HERNIA INCIDENCE
Analysis of late stomal complications following colon surgery[**16**] Mäkelä JT, Turku PH, Laitinen ST, 1997	27 %
A prospective audit of stomas-analysis of risk factors and complications and their management [**1**] Arum gam PJ, Bevan L, Macdonald L, Watkins AJ, 2003	51 %
Prospective analysis of stoma-related complications [**21**] Robertson I, Leung E, Hughes D, Spiers M, Donnelly L, Mackenzie I, 2005	40 %
Enterostomy Site Hernias: A Clinical and Computerized Tomographic Evaluation [**2**] Asım Cingi, Tebessum Cakir, Ali Sever and A. Ozdemir Aktan, 2006	52% clinical 78% CT
The prevalence of parastomal hernia after formation of an end colostomy [**18**] Servei de Cirurgia General i del Aparell Digestiu, Moreno-Matias J, Serra-Aracil X, Darnell-Martin A, Spain, 2008	47 %
Parastomal hernia treatment with prosthetic mesh repair [**6**] Department of Surgery and Perioperative Science, Umeå University, Sweden, 2010	45 %

Contributory factors are numerous and are related to the patient (obesity, smoking, effort), to the technique used (emergency intervention, the choice of size and position of the stoma), to the associated pathology (malnutrition, diabetes, tuberculosis, COPD and others), but most importantly, the evolution of the main consumptive disease [**7**]. Thus, due to the improvement of prognosis through adjuvant therapy, the life expectancy of neoplasia patients is continuously increasing, which in conjunction with the progressive occurrence of parastomal hernias during the evolution of the disease, mostly explaining the growing number of reported parastomal hernias. 

Parastomal hernia represents a complication that affects stomas on long-term, and after installation, hernia enlargement causes not only discomfort, but also impairs the attachment of stoma device, leading to intestinal prolapse and intestinal transit difficulties [**17**]. For this reason, the surgical treatment in often demanded by patients.

Conventional techniques of local repair are inappropriate considering the high recurrence rate, and the decision of stoma relocation depends on the associated pathology, which may counter-indicate general anesthesia, on previous surgical interventions, that are usually followed by a dense peritoneal adhesion syndrome [**5,19**]. Laparoscopic techniques have the advantage of a reduced morbidity and fast recovery, but they face the same counter-indications and the necessity of expensive dual-mesh materials [**10,12,13**]. 

With the increasing popularity of mesh repair of abdominal wall defects, many authors applied the same principle in parastomal hernia treatment. The downside was that of possible infection, due to the septic vicinity, which is prone to suppuration and, later, recurrence. 

The discovery of biocompatible, macro-porous, monofilament mesh, together with proper preoperative preparation, has minimized this risk [**20,22,23**]. 

**Fig. 1:**
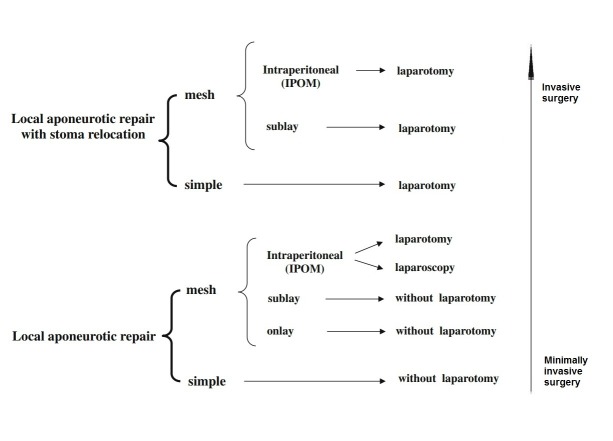
Local aponeurotic repair

Forced by the high incidence of parastomal hernia, some centers propose the use of alloplastic material in the construction of stoma, but they assume the relatively high price of this primary approach [**9,12,15**].

## Materials and methods

The purpose of this article is to make known a variant of alloplastic technique, without translocation, with a low degree of invasiveness, which can be performed successfully under spinal anesthesia, followed by a period of reduced hospitalization.

It is mainly addressed to patients with permanent stoma, often after amputation of the rectum, without signs of neoplasia, presenting with small and medium hernias.

In case of an elective surgery, preoperative preparation is possible, in order to combat associated metabolic deficiencies (hydro-mineral imbalance, anemia, hypoproteinemia), to prepare the colon (quantitative reduction of intestinal contents) and to decrease colonic septicity by using a preoperative antibiotic.

In the operating room, a Foley probe is inserted into the colostomy, the balloon is inflated in order to seal it and the entire field operator is covered by an iodized sterile drape in an effort to prevent further contamination of the wound and alloplastic material [**4**].

**Fig. 2:**
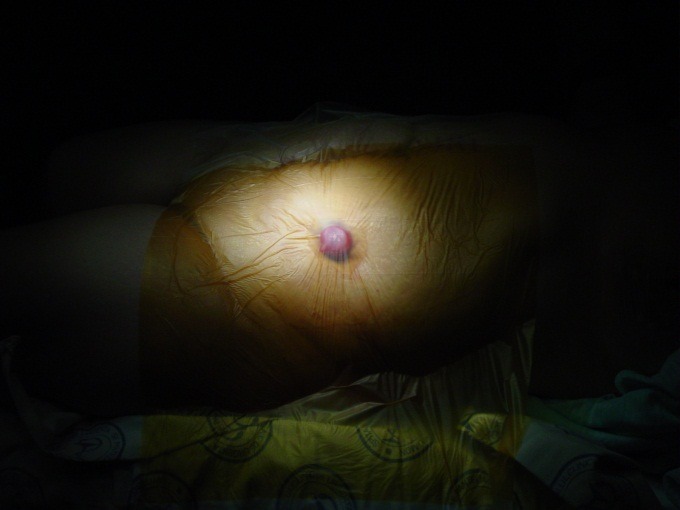
Local aponeurotic repair

**Fig. 3:**
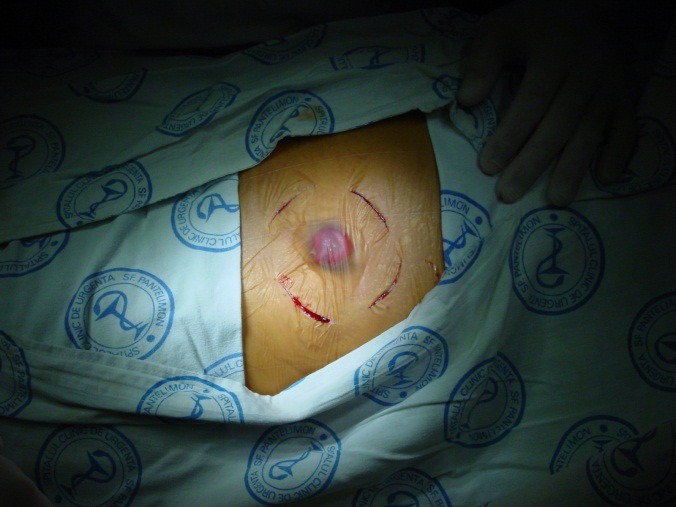
Local aponeurotic repair

Four semicircular incisions are made peristomal, at a distance, which allows postoperative stoma bag attachment. Next, subcutaneous dissection around the stoma is performed. Dissection is centripetal, until the reach of the colic wall, avoiding damage of the mesocolon, and centrifugal, distal of hernia sack. A circumferential area, ready for mesh placement is obtained.

**Fig. 4:**
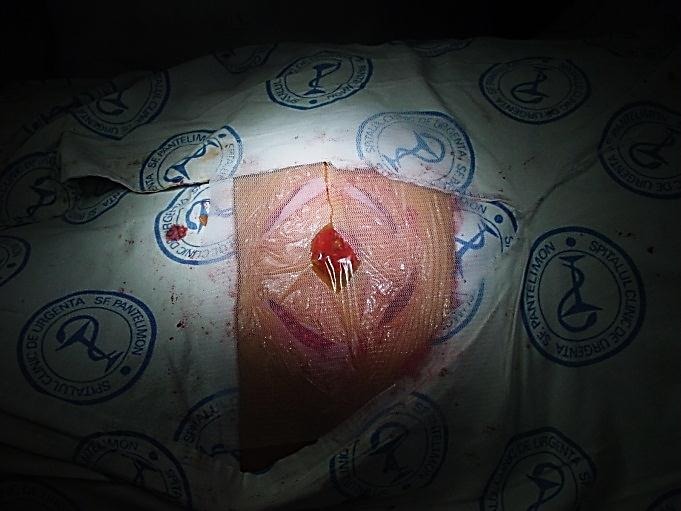
Local aponeurotic repair

**Fig. 5:**
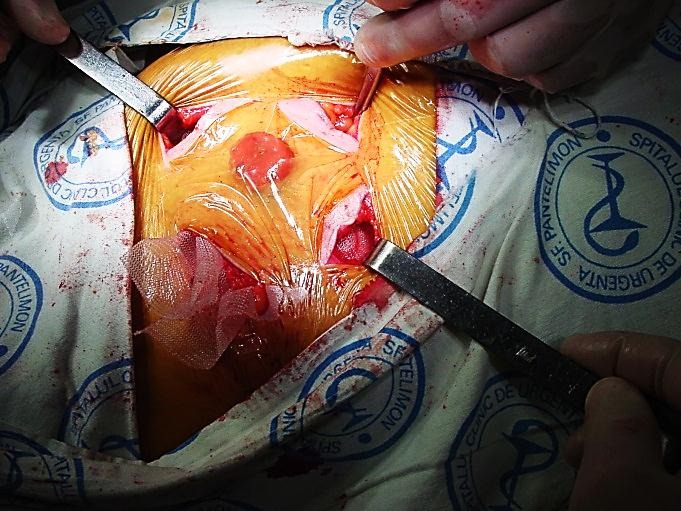
Local aponeurotic repair

The polypropylene mesh is prepared, so that the edges exceed by at least 5 cm the parietal defect border. The mesh is cut through on one side, to the central level, which creates four flaps approx. 2 cm long, which will be used to manufacture a collar of mesh around the infrategumentary portion of the colon. Newly created orifice is recalibrated so that it does not interfere with the transit through the colostomy [**4**].

The mesh is secured with interrupted sutures to the fascia and the initial gap is closed. To decrease the risk of infection, drainage is used not routinely, but may be necessary for obese patients, if extensive dissection is performed.

**Fig. 6:**
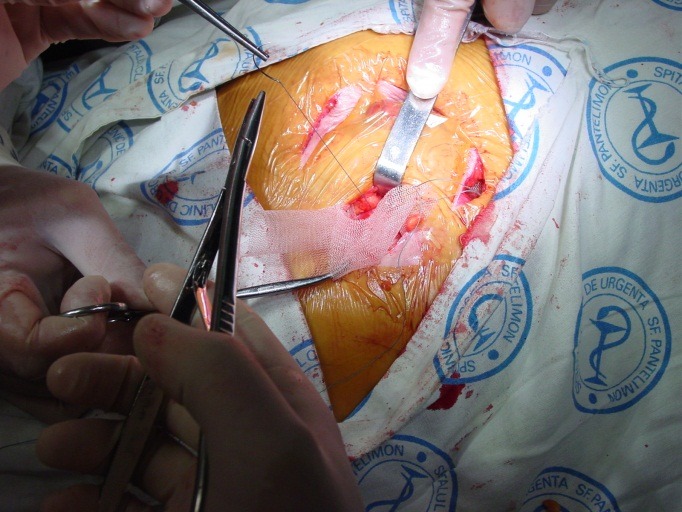
Local aponeurotic repair

**Fig. 7:**
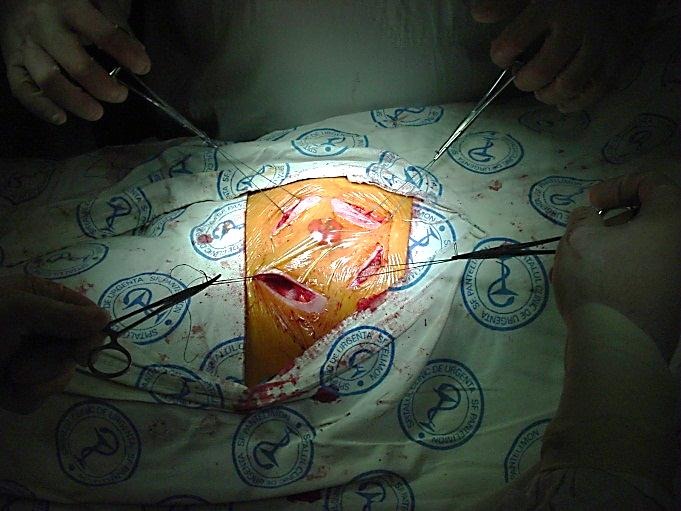
Local aponeurotic repair

The remotely incisions allow immediate prosthesis of colostomy, with early resumption of intestinal transit.

**Fig. 8:**
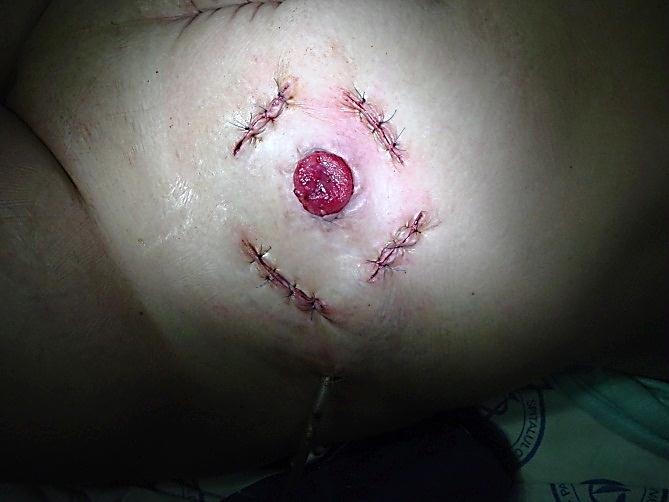
Local aponeurotic repair

**Fig. 9:**
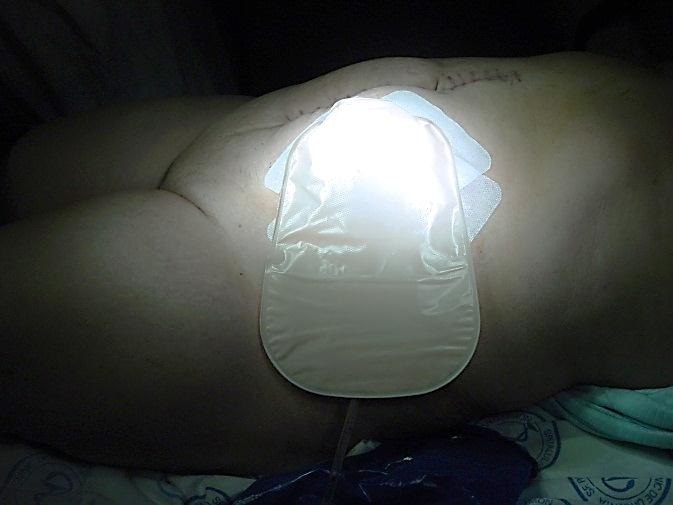
Local aponeurotic repair

The study group consisted of 6 patients with permanent left iliac anus (4 of them with previous Hartmann operation and 2 patients with abdominoperineal amputation of the rectum). Patients underwent these interventions one to three years prior to the occurrence of the parietal defect. No intra or postoperative complications occurred [**4**].

Patients were followed at 1 year and 2 years postoperatively and the results were favorable, with no relapses and improved quality of life through proper prosthesis of the stoma. This technique variation applies to small - medium parastomal hernias, in case of patients with permanent left iliac anus, with the declared intent of minimal invasiveness.

## Conclusions

1. Parastomal hernia complications are relatively rare, but the correct treatment plan is difficult to choose because it addresses debilitated patients with associated comorbidities.

2. Parastomal hernia is the most common complication that occurs to patients with permanent stomas and, by impairing the correct stoma prosthetics; it has a significant impact on quality of life.

3. Most alternative procedures open the peritoneal cavity, with all the risks inherent to adhesion dissection and / or stoma translocation.

4. The suggested procedure offers multiple advantages, resulting from the minimum invasive nature, the absence of risks associated to general anesthesia and laparatomy, and their negative impact for these types of patients.

5. Similar to alloplastic treatment results obtained for the other abdominal wall defects, the use of mesh provides a good quality, tension free prosthesis, with a low risk of recurrence.

6. The initial (ideal) location of stoma is preserved, by maximizing the comfort of future prosthesis maintenance.

7. The intervention cost is kept at a low level by the use of regular alloplastic materials, the short period of convalescence allowing fast social reinsertion [**24**].

8. At the same time, the possibility of one-day surgery for these patients can be taken into consideration.

9. However, a frequently used procedure in general surgery clinics, colostomy, should not be considered a handicap, by providing an appropriate psycho-socio-medical environment. 

10. Efforts for decreasing the number of patients who need permanent colostomy are required, through both precocious screening of malignant colorectal and genital conditions and also by applying appropriate, well standardized, diagnostic and treatment.
